# Effects of Thermal Pre-Treatments and Drying Processes on the Retention of Phytonutrients, Vitamins, and Antioxidant Activity in Dried Okra (*Abelmoschus esculentus* L.)

**DOI:** 10.3390/foods15020311

**Published:** 2026-01-15

**Authors:** Megan L. Reid-Fitten, Corrie P. Cotton, Byungrok R. Min, Caleb I. Nindo, Zachary F. Williams

**Affiliations:** Department of Agriculture, Food and Resource Sciences, University of Maryland Eastern Shore, Princess Anne, MD 21853, USA; bmin@umes.edu (B.R.M.); cinindo@umes.edu (C.I.N.);

**Keywords:** okra, processing, preservation, phytonutrients, antioxidant activity, flavonoids, vitamins

## Abstract

Opportunities to capture anticipated niche markets for diverse populations continue to rise. Okra (*Abelmoschus esculentus* L.), considered a high-value crop, is rich in nutritional and medicinal properties; however, fresh okra is highly perishable. This study examined the effects of thermal pre-treatments and drying processes in combination on the nutritional quality of dried okra. The experiment consisted of two thermal treatments (steam-blanched and hot water-blanched, and the control) and three drying treatments (freeze-dried, hot air-dried, and infrared-dried). Okra was grown in black plastic mulch, harvested twice per week, and processed three times throughout the growing season. The study analyzed moisture content, water activity, phytonutrients, ascorbic acid, β-carotene, and antioxidant activities. No significant differences were observed in moisture content and water activity among the treatments. Significant differences were observed among treatments and harvest time for total phenolic and flavonoid contents and antioxidant activity. Notable differences in β-carotene content were observed across all treatments. Based on the findings, the steam-blanched freeze-dried treatment was the most effective preservation technique for maintaining the nutritional and functional quality of dried okra. Hot water-blanching, hot air-drying, and infrared-drying were the least effective for the development of a high-value, nutrient-dense dried okra value-added product.

## 1. Introduction

In the Delmarva region of the United States, economic opportunities exist to increase the production of specialty crops catering to ethnically diverse consumers [1]. The growth of the diverse population in the Mid-Atlantic region has created a strong demand for crops and products that will meet their needs. Opportunities to capture anticipated niche markets for ethnic crops will continue to grow. Therefore, local farmers must adapt to the development of new crops and create value-added opportunities to maintain economic value and extend the appropriate year-round supply of ethnic crops to the area [1].

One of the ethnic crops in high demand in the Mid-Atlantic region is okra (*Abelmoschus esculentus* L.). Okra is an annual herbaceous plant that originated in Ethiopia and Sudan, and was propagated in North African countries, the Mediterranean, Arabia, and India [2,3]. It is considered an economically important vegetable grown in the sub-tropical, tropical regions, and warm temperate climates in different countries such as Africa, Asia, South Europe, and America [4,5,6]. Okra is considered one of the Malvaceae family’s most widely known and utilized species. It is known by several names in different countries, such as lady finger in England, gumbo in the United States of America, quiabo in Portuguese, and bhindi in India [2,4,5,7]. Okra is consumed as a vegetable, used in salads, stews, and soups, and is often eaten fresh, dried, or boiled [5]. The immature pods of the vegetable are used to make pickles, and the water-soluble polysaccharides from okra are used in ice cream and baked products.

Okra also plays a significant role in human health and is rich in nutrients, such as proteins, free amino acids, antioxidants, vitamins, trace elements, and dietary fiber [4,6]. Due to its low fat and carbohydrate contents, okra is considered a low-calorie and cholesterol-free healthy vegetable. In addition to its nutritional values and functions, okra is also suitable for medicinal uses, and its consumption has been associated with improvements in glycemic control and lipid profiles, as well as anti-inflammatory effects, suggesting potential health benefits [5].

Despite being a good source of nutrients, okra is highly perishable due to its high moisture content and respiration activities. Fresh okra has a high moisture content of 88–90% (wet basis), and safe storage typically requires reducing the moisture content to around 10% (wet basis) [6]. Drying is a process of simultaneous heat and mass transfer where the heat is applied to the product, which increases the product’s temperature and vaporizes the moisture. The drying process helps reduce the weight and volume for storage and transportation costs, and extends the food product’s storability [8]. Drying extends the shelf life of fruits and vegetables by preventing the growth of microbes and reducing enzymatic reactions by lowering the water activity [9]. Previous studies on okra have shown that blanching can inactivate degradative enzymes, helping to preserve phenolic compounds, vitamin C, and antioxidant capacity during subsequent drying [10]. Different drying methods, including hot air, infrared, and freeze-drying, have been reported to influence the retention of bioactive compounds, with freeze-drying generally providing the highest retention of phenolics and antioxidant capacity, while hot air and infrared-drying can lead to moderate losses depending on temperature and exposure time [11,12]. These findings highlight the importance of selecting appropriate pre-treatment and drying conditions to maximize the nutritional and functional quality of okra.

Several drying methods are used to improve food products’ shelf life and storage stability. However, some drying techniques can result in the degradation of phyto-constituents due to their thermal sensitivity [13]. It is vital to select the appropriate drying methods to retain appearance, aroma, and nutritional content, and to prevent spoilage caused by microorganisms and enzymes responsible for undesired chemical changes in the dried food products. Several drying methods used to preserve fruits and vegetables include freeze, hot air, infrared, sun, and microwave drying [9,13,14]. However, each drying method comes with its advantages and limitations. It is hypothesized that blanching may reduce phenolic content and antioxidant capacities due to leaching, while freeze-drying is expected to best preserve bioactive compounds compared with hot air and infrared-drying [15]. The freeze-drying method can preserve the original properties, such as the color and shape, but it consumes extensive time and energy. Although freeze-dried products are known to be of good quality, the efficiency of the freeze-drying method is known to be low [16,17]. Hot air-drying is a low-cost conventional method, but the drying duration can cause the degradation of nutritional compounds, color, and shape. It also has a negative impact on the nutrient quality, flavor, and texture of the products [18,19]. As for the infrared-drying method, it can preserve nutrients similar to the freeze-drying method, but it is also known to be expensive, and consumes much energy, with most of the radiant heat being applied on the product’s surface [15].

Fresh okra is considered highly perishable and can only last a few days after harvesting at room temperature or in the refrigerator. Okra is mainly sold fresh and frozen in the market. However, due to its nutritional benefits and its demand by the growing, diverse population in the region, the need to extend the shelf life and create value-added products has gained much attention. Hence, the opportunity presents itself to explore the market of dried okra with an extended shelf life. Therefore, the objective of this study was to evaluate harvest time and the combined effects of steam and hot water-blanching and three drying methods (freeze, hot air, and infrared) on the moisture content, water activity, phenolics, flavonoids, antioxidant capacity, and vitamins A and C in dried okra value-added products.

## 2. Materials and Methods

### 2.1. Crop Production

#### 2.1.1. Plant Growth and Development

In March 2022, Clemson Spineless OG okra seeds (Johnny’s Selected Seeds, Winslow, ME, USA) were sown in 50-cell seedling trays containing Pro-mix Bx (Premier Horticulture Inc., Quakertown, PA, USA). Seeds were sown in a ventilated controlled greenhouse during the spring, with daytime temperatures maintained at 75–95 °F (24–35 °C) and nighttime temperatures at 60–75 °F (15–24 °C). As needed, the seedlings were fertilized with Jack’s Classic All-Purpose 20-20-20 (J.R. Peters, Inc., Allentown, PA, USA). The okra seedlings were transplanted 45 cm apart into black plastic mulch rows, with 1 m spacing between rows, at the University of Maryland Eastern Shore (UMES) Agricultural Experiment Station in Princess Anne, Maryland, which experiences a warm temperature to subtropical climate during the summer. The soil at the site is classified as Quindocqua silt loam with approximately 45% drainage capacity and a typical pH of 6.0–6.8, conditions suitable for okra cultivation. During the growing period (March to September 2022), average daytime temperatures ranged from 75 to 95 °F (24–35 °C), which is within the optimal range for okra growth. Nighttime temperatures generally remained above 60 °F (15 °C). The okra plants were irrigated and fertilized bi-weekly through drip irrigation and grown according to conventional agricultural practices. The experiment began once the okra was harvested. The experimental design consisted of nine treatments throughout the study. The replications of the study were independent processing batches.

#### 2.1.2. Harvesting and Sample Preparation

The standard commercial maturity for okra pods is when they reach 8 to 12 cm in length, 1.5 to 2.0 cm in diameter, and exhibit a firm yet tender texture. Okra was harvested twice per week and processed three times throughout the growing season. The first harvest was at the beginning of the growing season (56 days after transplanting (DAT)), the second harvest was in the middle of the growing season (84 DAT), and the third harvest was at the end of the growing season (114 DAT). Okra pods showing signs of senescence or excessive fibrousness were excluded from the study. These criteria ensure consistent and reproducible maturity in comparison with other studies. Harvested okra was placed into 14-gallon plastic tubs and transported to the lab. The okra was cleaned, washed with running tap water, drained, dried with paper towels, and stored in a refrigerator at 4 °C until processing. The pods remained whole to prevent the loss of mucilage during thermal processing.

### 2.2. Experimental Design

The experimental design consisted of two thermal pre-treatments (steam-blanch (SB) and hot water-blanch (HWB)), a control (C), and three drying treatments (freeze-dried (FD), hot air-dried (HAD), and infrared-dried (ID)) in combinations, resulting in a total of nine treatments: T1: C-FD, T2: C-HAD, T3: C-ID, T4: SB-FD, T5: SB-HAD, T6: SB-ID, T7: HWB-FD, T8: HWB-HAD, and T9: HWB-ID. All treatments were triplicated. Samples dried without any pre-treatment served as the control (C). Although the okra was harvested weekly, the thermal processing and drying were only conducted three times throughout the growing season (at the beginning, in the middle, and at the end). All nine processing treatments were applied at each harvest time.

### 2.3. Thermal Processing

#### 2.3.1. Steam-Blanching

A stainless-steel tray was used to transport an appropriate amount of the samples into the blanching–cooling chamber (Dixie M-6 Steam Blancher-cooler, Athens, GA, USA). Blanching was performed on whole okra pods to minimize mucilage loss. The steam-blanching process was conducted with the boiler steam pressure at 40 PSI, and the temperature inside the blanching chamber was approximately 100–110 °C with the hood closed. Each batch contained an appropriate amount of okra arranged on a single-layer stainless-steel tray. The product core reached the target temperature in approximately 3 min, after which the samples were cooled immediately by spraying cold water over the samples for 3 min. Samples remained on the stainless-steel trays to drain the excess water.

#### 2.3.2. Hot Water-Blanching

The hot water-blanching process was conducted by first heating water to 98–100 °C in a 25-gallon steam kettle (Vulcan, Baltimore, MD, USA). The okra pods were blanched whole at approximately 98–100 °C for 3 min using a water-to-sample ratio of about 3 gallons per 950 g of okra. The water was continuously stirred during blanching to ensure uniform heat transfer. The hot water-blanched okra was immediately cooled by immersion in an ice bath, assumed to be approximately 0 °C. Cooling was sufficient to reduce the product core temperature to below <10 °C within 3 min, based on standard blanching and cooling procedures. The samples were then spread evenly on a stainless-steel sieve to drain the excess water.

#### 2.3.3. Slicing

The fresh (control) and thermally processed okra were thinly sliced into approximately 3–5 mm cross-sections, and slices were cut consistently to ensure uniform thickness, as slice size and orientation strongly influence the drying kinetics and nutrient retention. Samples were packaged in double Ziploc bags, labeled, and stored in a −80 °C freezer prior to the drying process. The samples’ storage duration was recorded between 24 and 72 h; all treatments were processed for drying in close succession to minimize potential confounding effects from differences in frozen storage. The fresh andblanched okra slices were divided evenly into three appropriate portion sizes before the drying (freeze, hot air, and infrared) process.

### 2.4. Drying

This study used three drying treatments to dry the fresh, steam-blanched, and hot water-blanched okra. The fresh and blanched okra was evenly divided into three and dried using the freeze, hot air, and infrared-drying methods.

#### 2.4.1. Freeze-Drying

Samples were freeze-dried using a benchtop freeze dryer (Harvest Right, North Salt Lake, UT, USA). The key operating parameters included a condenser temperature of 125 °C, while the chamber pressure was not recorded; the shelf/tray temperature was 135 °F. The thermally processed samples were frozen for about 24–72 h at −80 °C to ensure complete freezing of water. This pre-freezing step stabilizes the sample structure, facilitates uniform sublimation during the primary drying, and helps preserve phytochemicals and other sensitive compounds. The fresh and blanched sliced okra samples were evenly spread on metal trays lined with aluminum foil and freeze-dried for approximately 24–36 h, depending on the weight of the samples. These conditions were applied consistently across all treatments to ensure reproducibility.

#### 2.4.2. Hot Air-Drying

A food dehydrator (Model LT-19, 16 Tray, Dryer, Zhejiang Lianteng Machinery Co., Ltd., Wenzhou, Zhejiang Province, China), consisting of a centrifugal fan to supply airflow at 0.3 m/s and a volumetric flow of 100–300 L/min, an electrical heater, an air filter, and a proportional temperature and time controller, was constantly applied across all hot air-dried treatment. The sliced samples were evenly distributed on several drying mats and placed on 8 × 10 stainless-steel wire mesh trays, and dried at 50 °C. Samples took approximately 18 h for the control and 8–12 h for the steam and hot water-blanched samples to dry.

#### 2.4.3. Infrared-Drying

An infrared dryer was custom-built using plexiglass and infrared lamps (Nemco 6150-48-CP, Hicksville, OH, USA) with a power level of 75–83 W. The infrared lamps (2 total) were used as the heat source; the exact wavelength range was not specified. The lamps, generating heated air between 55-60 °C (taken at different intervals and monitored with a non-contact infrared thermometer), were positioned at a distance of about 6-8 inches from the samples’ surface and a small in-built fan supplying the airflow (velocity was not recorded). Vents were provided on the sides of a glass enclosure, and because the okra was in small quantities, spread out uniformly on a drying mat, and then placed on a wire mesh, natural convection was adequate to remove the evaporating moisture. Small temperature fluctuations occurred across the trays, which may have contributed to less uniform air flow patterns than that of a forced-convection commercial system. Samples were dried for 18–24 h for the control and 8–12 h for the steam and hot water-blanched okra. Both hot air and infrared-dried okra samples were drawn at 1 h intervals for moisture content, water activity, and sample weight measured using a digital balance. Drying was completed when the moisture content of the hot air-dried and infrared-dried samples was approximately 10% (wb), and the water activity was within the 0.30–0.35 range. These conditions were maintained consistently across all treatments to ensure reproducibility.

#### 2.4.4. Moisture Content

A Halogen Infrared Moisture Analyzer (HB43-S, Mettler Toledo-AG, Zurich, Switzerland) was used to determine the fresh and dried okra moisture content (%). The samples were dried until the final moisture content reached a level of less than 10%.

#### 2.4.5. Water Activity

Aqua Lab CX-2 (METER Group, Pullman, WA, USA) was used to determine the water activity (a_w_) of okra samples drawn from the dryer at 1 h intervals until the a_w_ at 25 °C had reached the desired value (0.30–0.35 range).

### 2.5. Analysis

#### 2.5.1. Sample Preparation

Dried okra samples (nine treatments with three replications) weighed between 50 g and 75 g. The powder products were obtained by individually grinding the weighed samples in a milling machine (Vetch Mill, Fisher Hamilton, Newtown, PA, USA) with a 0.5 mm sieve. The ground products were weighed, placed into double Ziploc bags, and stored in a freezer at −80 °C until further analysis.

#### 2.5.2. Extraction of Phenolic Compounds

Methanolic (MeOH) extract was prepared according to [20] to analyze total phenolic and flavonoid contents and antioxidant capacities. Phenolic compounds were extracted from 0.25 g of okra in 5 mL of 80% methanol (MeOH) extraction, performed twice for a total of 10 mL. This solvent-to-sample ratio is sufficient for complete extraction of phenolics from okra pods and other fibrous plant matrices [21]. The mixture was homogenized for 30 s using a homogenizer (Polytron PT 10-35 GT, Kinematica Ag, St. Moreno Valley, CA, USA) and then agitated at 300 revolutions per minute (rpm) for 2 h at room temperature (25 °C) in a platform shaker (Inova 2000, New Brunswick Scientific, Edison, NJ, USA). After incubation, the tubes were centrifuged at 3000× *g* for 30 min at 4 °C. After centrifugation, the supernatant was transferred to 15 mL polypropylene centrifuge-labeled tubes. Another 5 mL of 80% methanol MeOH was added to the pellet, and the extraction procedure was repeated. The supernatant of each sample was pooled and stored in a freezer at −18 °C.

##### Determination of Total Phenolic Content (TPC)

TPC was determined using the Folin–Ciocalteu spectrophotometric method, as outlined in [20]. After appropriate dilution, 50 µL of the diluted extract was mixed with 550 µL distilled deionized water (DDW) and 250 µL of 20% Folin–Ciocalteu reagent. The mixture was allowed to stand for 5 min at room temperature. Then, 500 µL of 0.5 M ethanolamine was added to the mixture and set to stand for 90 min at room temperature for color development. After color development, the absorbance was measured at 760 nm against the reagent blank using a spectrophotometer (UV-2450, Agilent, Tokyo, Japan). A series of concentrations of gallic acid solution in methanol (25, 50, 100, 150, and 200 µg gallic acid/mL) was used to prepare a standard curve to determine the total phenolic content, which is expressed as mg gallic acid equivalents (GAE)/g sample dry weight (DW).

##### Determination of Total Flavonoid Content (TFC)

TFC was analyzed using the spectrophotometer method described by [20]. After an appropriate dilution, 100 µL of the diluted extract was mixed with 500 µL DDW and 40 µL of 5% sodium nitrate (NaNO_2_, *w*/*v*) solution, and the mixture was allowed to stand for 5 min at room temperature. Then, the mixture was mixed with 75 µL of 10% aluminum chloride (A1C1_3_, *w*/*v*) solution and allowed to stand for another 6 min at room temperature. The mixture was then mixed with 250 µL of 1 M sodium hydroxide (NaOH) solution. The final mixture was centrifuged at 12,000× *g* for 2 min at room temperature. The absorbance was measured against a blank at 510 nm. The standard was prepared using a series of (+)-catechin solutions in methanol (25, 50, 100, 150, and 200 µg (+) catechin/mL MeOH) to determine the total flavonoid content, which is expressed as mg (+)-catechin equivalents (CE)/g sample DW.

#### 2.5.3. Determination of Antioxidant Capacity

Oxygen radical absorbance capacity (ORAC) and 1,1 dipheny1-2-picrylhydrazyl radical scavenging capacity (DPPH) were determined to assess the antioxidant capacities of okra samples. Both assays were conducted according to the procedure established by [20].

##### Oxygen Radical Absorbance Capacity (ORAC) Method

The ORAC assay was conducted as described by [20] using a microplate reader equipped with fluorescence intensity detection and auto-injectors (FLOUstar Omega BMG Labtech, Ortenberg, Germany). After appropriate dilution with 0.075 M phosphate buffer (pH 7), 25 µL of the supernatants and 150 µL of fluorescein solution of the phosphate buffer (final con. 67.95 µM) were added to all wells of a 96-well black polystyrene flat-bottom microplate (Corning, Corning, NY, USA). The plate was covered and incubated in the microplate reader for 10 min at 37 °C. After the incubation, 25 μL of AAPH (2,2′-azobis (2-amidinopropane) dihydrochloride) solution prepared in cold phosphate buffer was added to each well via an auto-injector to make its final concentration of 37.71 mM. Immediately after the injection, fluorescence responses were recorded at the excitation wavelength of 485 nm and an emission wavelength of 520 nm at 2 min intervals for 38 min at 37 °C to draw a fluorescence decay curve. The plate was shaken for 8 s. The area under the curve (AUC) was calculated as follows: AUC = (0.5 + f2/f1 + f3/f1 … + fi/fl) × CT, where f1 is the first fluorescence reading, fi is the fluorescence reading at cycle i, and CT is the cycle time in minutes. A series of Trolox (6-hydroxy-2, 5, 7, 8-tetramethylchroman-2-carboxylic acid) standard solutions (6.25–50 µM) was prepared to generate the standard curve against their AUC. The ORAC results were expressed as μmol Trolox equivalent per gram dry weight sample (µmol TE/g DW).

##### DPPH Radical Scavenging Capacity Method

The DPPH radical scavenging capacity was conducted as described by [20]. An appropriate amount of dilution was used to determine the DPPH for each sample. An amount of 50 µL of the supernatant was mixed with 900 µL DPPH solution (40 mg/L in 100% methanol solution) in a 96-well template plate. To avoid exposure to light, the template was completely covered in aluminum foil, and samples were shaken at 300 rpm for 30 min at room temperature. Subsequently, 200 µL of the reaction solution was transferred to a corresponding 96-well microplate, and the absorbance was determined at 515 nm using a microplate reader. The DPPH radical scavenging capacity was calculated using a calibration curve, developed from a series of Trolox solutions (6.25–50 µg/mL in 100% MeOH), and was expressed as mg TE/g sample (DW).

#### 2.5.4. Vitamin C (Ascorbic Acid) and Vitamin A (Beta-Carotene) Tests

Twenty-five grams of dried ground samples were sent to Medallion Labs (Plymouth Ave., Minneapolis, MN 55427, USA) for vitamin A and C analysis. Vitamins were analyzed only from the second harvest (84 DAT) due to resource constraints. All analytical procedures were obtained directly from the Medallion Labs website [22]. Briefly, ascorbic acid (vitamin C) was extracted using a methanol/metaphosphoric acid solution and converted to dehydroascorbic acid, which reacts with ortho-phenylenediamine to form a fluorophore. The fluorescence intensities against a standard curve were used to determine the total vitamin C content (mg/100 g DW). A blank was simultaneously run on each sample to determine the level of background fluorescence. Vitamin A analysis included quantification of alpha, cis-beta, trans-beta, total beta, and total carotene. After enzymatic digestion, the sample was homogenized with tetrahydrofuran to extract the carotenoids. The extract was filtered and analyzed using reverse-phase HPLC with UV/Vis detection to quantify carotenoids (µg RAE/100 g DW (retinol activity equivalents)). All quantified data were acquired directly from Medallion Labs and interpreted by the authors.

### 2.6. Statistical Analysis

Analysis of variance (ANOVA) was performed using the Statistix 9.0 application. Harvest time was not treated as a fixed factor, and no blocking or random effects were included in the model, as all treatments were applied uniformly across the experimental site. Differences among treatment means were compared using Tukey’s honest significant difference (HSD) test at the 0.05 significance level. Data are presented as mean ± standard deviation (SD) of the replicates.

## 3. Results

### 3.1. Moisture Content and Water Activity

It has been observed that okra is highly perishable due to a high moisture content of 88% to 90% (*w*/*w*), and that 10% was the safe moisture level for okra storage [23]. Water activity (a_w_) is considered one of the key factors in microbial growth and enzymatic and nonenzymatic reactions. Water activity is an indicator of the availability of free water in a food product that can participate in biological processes, such as microbial growth, as well as chemical reactions [24,25,26]. Table 1 shows the moisture content (%) and water activity (a_w_) of okra as affected by thermal pre-treatments and drying processes. Although the moisture content (3.16–6.72%) and water activity (0.29–0.44) values show some numerical variation among treatments, ANOVA indicated that these differences were not statistically significant (*p* > 0.05). Thus, the observed variability reflects natural experimental variation rather than treatment effects. The moisture content of the treatments fell within the required safe moisture threshold for dried okra storage, which is lower than 10% [23].

The a_w_ among treatments fell within the range of 0.29 to 0.44. The a_w_ < 0.6 can inhibit the growth of spoilage microorganisms as most bacteria cannot grow below an a_w_ of 0.90 [25]. However, in this study, most dried okra samples exhibited water activity below 0.35; several samples were ≥0.35; and one treatment reached higher levels up to 0.44. This prevents the growth of spoilage microorganisms, which is generally considered favorable for shelf stability [25]. The shelf life of dried products depends on the obtained water activity level and the storage conditions (room temperature and storage package) [27]. They later investigated the effect of pre-treatment in ethyl alcohol pre-treatments (5, 15, 60, and 180 s) combined with the application of ultrasound on the convective drying behavior and quality properties of carrot tissue directly after the treatment and after the drying process [27]. These findings on how pre-treatments influence moisture removal and tissue structure in carrots provide a useful context for understanding similar drying mechanisms in okra, although their study did not include okra slices.

### 3.2. Phenolic Content and Antioxidant Capacity of Dried Okra

The analysis of variance revealed that all evaluated parameters differed significantly regardless of the treatments applied. Significant differences (*p* < 0.05) of thermal pre-treatments, drying processes, and harvest times were observed in phenolic contents and antioxidant capacities in okra.

#### 3.2.1. Total Phenolic Content (TPC)

Table 2 shows the total phenolic contents in dried okra (mg GAE/g DW) as affected by the thermal pre-treatments, drying processes, and harvest times. The C-FD treatment showed a significantly higher TPC at the first and third harvests, with levels of 7.92 ± 0.3 and 8.62 ± 0.4 mg GAE/g, respectively, compared to the other treatments. The C-FD and C-HAD treatments showed a significantly (*p* < 0.05) higher TPC in the second harvest (84 DAT) with a level of 8.23 ± 0.9 and 8.25 ± 0.5 mg GAE/g DW, respectively, compared to the other treatments. When comparing harvest times, TPC was significantly higher (*p* < 0.05) in the second harvest for all treatments except HWB-FD (5.04 ± 0.1 mg GAE/g (DW)). These results suggest that the extract has potential as a source of beneficial phenolic compounds, which aligns with other reports on the phenolic content of okra [28,29,30].

The results show that the TPC was significantly affected by the treatments. The phenolic contents of the okra decreased after blanching, regardless of the drying method applied. The lower phenolic content may be due to the leaching of water-soluble phenols after tissue softening of the okra. These findings were similar to those reported by others [10,31,32]. It has been reported that the TPC might be affected by the processing method, drying temperature, as well as environmental factors such as the time of harvest [33,34]. The results shown in Table 2 indicate that blanching, both steam (SB) and hot water (HWB), significantly reduced the TPC across all drying methods compared with the corresponding unblanched controls. For example, in the first harvest, the freeze-dried SB and HWB samples had TPC values of 4.77 ± 0.2 and 6.65 ± 0.4 mg GAE/g DW, respectively, compared with 7.92 ± 0.3 mg GAE/g DW in the freeze-dried control (C-FD), corresponding to reductions of approximately 40% and 16%. Similar reductions were observed for hot air and infrared drying, where blanching resulted in TPC losses ranging from approximately 15 to 45% relative to the corresponding controls, depending on the pre-treatment and harvest. Freeze-dried controls consistently retained the highest TPC across harvests (7.92 ± 0.3–8.62 ± 0.4 mg GAE/g DW), whereas hot air and infrared-drying generally resulted in moderate reductions in TPC. These decreases following blanching are likely associated with phenolic leaching and thermal degradation; however, the significant interaction with harvest time indicates that treatment effects are harvest-dependent, which were similar to the results of studies on the influence of drying methods on the physicochemical properties of okra [4,23].

#### 3.2.2. Total Flavonoid Content (TFC)

Table 3 presents the level of TFC of dried okra (mg CE/g DW) subjected to different pre-treatment and drying combinations. At the first harvest, unblanched control treatments exhibited a higher TFC than most blanched treatments, consistent with the trend observed for TPC. Specifically, C-FD (2.32 ± 0.03 mg CE/g DW) and C-HAD (2.26 ± 0.03 mg CE/g DW) showed significantly higher (*p* ˂ 0.05) flavonoid levels than steam-blanched and hot water-blanched samples, particularly SB-FD (1.22 ± 0.03 mg CE/g DW) and SB-HAD (1.49 ± 0.03 mg CE/g DW), corresponding to reductions of approximately 47% and 34%, respectively, relative to C-FD. Hot water-blanching also resulted in a lower TFC compared with the controls, although the magnitude of reduction was generally less pronounced than for steam-blanching. The reduction in TFC following blanching is likely attributable to leaching water-soluble flavonoids into the blanching medium and oxidative degradation induced by thermal exposure [10,35].

In later harvests, treatment rankings varied with both pre-treatment and drying methods, indicating that the effects of processing on TFC were not consistent across harvest times. At the second harvest, C-ID showed a significantly higher TFC (2.17 ± 0.1 mg CE/g DW) compared with other treatments. The C-HAD and C-ID treatments showed a significantly higher TFC at the third harvest (2.47 ± 0.1 and 2.30 ± 0.01 mg CE/g DW, respectively) when compared to the blanched samples. When comparing harvest times, TFC was significantly higher at the first harvest for most treatments, except the C-ID, SB-FD, and SB-ID treatments, which displayed lower flavonoid levels of (2.12 ± 0.1, 1.22 ± 0.03, and 1.55 ± 0.01 mg CE/g DW, respectively). Across treatments, TFC tended to be higher at the first and second harvests than at the third harvest; however, the magnitude of this effect depended on the pre-treatment and drying method. In general, early- to mid-season harvests showed greater flavonoid retention, while late-season harvests were associated with a lower TFC, particularly in blanched samples.

Overall, steam and hot water-blanching methods applied to okra induced noteworthy alterations in the TFC of dried okra, compared to the control treatments. A similar study was conducted by [10], which also showed that TPC and TFC decreased based on the application of heat. The observed reductions in the flavonoid content of okra may be attributed to several factors, such as the drying temperature, leaching of some water-soluble flavonoids during blanching, and enzymatic oxidation [10,35]. These results demonstrate that the pre-treatment, drying method, and harvest time interact in a complex manner to influence TFC, requiring consideration of specific treatment combinations within each harvest.

#### 3.2.3. DPPH Radical Scavenging Capacity (DPPH)

The results of the DPPH radical scavenging capacity (mg TE/g DW) in dried okra are shown in Table 4. The C-FD and C-HAD treatments showed a significantly higher (*p* < 0.05) DPPH concentration at the first harvest (16.44 ± 0.78 and 15.58 ± 0.67 mg TE/g (DW), respectively) and the second harvest (16.23 ± 1.64 and 17.04 ± 1.93 mg TE/g DW, respectively), while C-HAD and C-ID were significantly higher (*p* < 0.05) at the third harvest (14.58 ± 0.64 and 14.52 ± 0.75 mg TE/g DW, respectively) when compared to the other treatments. The DPPH concentration was significantly higher (*p* < 0.05) in the first and second harvests than in the third harvest for most treatments, except for C-HAD and SB-ID. Similar results were reported by [10], who found that fried okra showed a significant decrease in DPPH inhibition activity, which may be due to the destruction of polyphenols and other antioxidant compounds during the preparation process or extended frying time. No significant differences were observed for the C-HAD and SB-ID treatments when compared to the other harvest times.

The DPPH in steam and hot water-blanched okra samples were significantly lower (*p* < 0.05) than the controls, probably due to the thermal pre-treatments. It has been stated that the reduction in antioxidant activity may be related to a loss of total phenolic content because of the oxidation and polymerization of phenolic compounds, which are known to be the main compounds responsible for the antioxidant activity of plants [10]. The results in this study showed that the controls (C) without the thermal pre-treatment were rich in phenolic compounds with a higher DPPH concentration compared to the thermal pre-treated samples, regardless of the harvest time. These results indicate that the okra extract contains an antioxidant compound, which aligns with other studies on the antioxidant activity of Spirogyra and okra [23,28,29,36].

#### 3.2.4. Oxygen Radical Absorption Capacity (ORAC)

The results of the ORAC capacity (µmol TE/g DW) in dried okra are shown in Table 5. The SB-ID, HWB-FD, and HWB-ID treatments were significantly higher (*p* ˂ 0.05) in ORAC in the first harvest with 9.39 ± 0.29, 10.08 ± 0.93, and 9.50 ± 0.57 µmol TE/g DW, respectively. Control-ID was significantly higher (*p* ˂ 0.05) in the second harvest (12.30 ± 0.53 µmol TE/g DW). C-HAD and C-ID (14.71 ± 1.22 and 14.91 ± 0.83 µmol TE/g DW, respectively) treatments were significantly higher at the third harvest when compared to the other treatments (Table 5). The ORAC concentration was significantly higher at the second and third harvests for most treatments. No significant differences were observed for the HWB-FD and HWB-ID treatments when compared to the other harvest times.

Differences between ORAC and DPPH arise from their distinct mechanisms; DPPH measures electron-donating capacity, whereas ORAC is based on hydrogen atom transfer and is more sensitive to chain-breaking antioxidants and certain phenolic subclasses. Consequently, some treatments, such as C-ID samples at later harvests, showed higher ORAC values despite moderate DPPH activity, reflecting the selective retention or extractability of compounds detected by ORAC. These assay-dependent differences have been widely reported in thermally processed plant foods and reflect variations in antioxidant composition rather than methodological inconsistencies [37,38].

The relationships between harvest time and the phenolic contents (TPC and TFC) as well as antioxidant capacities (DPPH and ORAC) of okra are presented in Table 2, Table 3, Table 4 and Table 5. The results demonstrated that the phenolic compound capacities were significantly influenced by the harvest time. Significant differences (*p* < 0.05) were observed in the first and second harvests, during which the TPC and TFC levels were significantly influenced by thermal pre-treatment, drying methods, and harvest times, with clear interactions among these parameters. TPC and TFC generally declined by the third harvest for most treatments, although some exceptions occurred depending on the pre-treatment and drying. These results are consistent with a study conducted by [39], where they too saw an increase in the TFC of okra during the fruit maturation stage, which then decreased during harvest. In many treatments, the TFC was highest at the earliest harvest, which contrasts with [39], who found that the flavonoid content increased during maturation before declining. The key contrast lies in the sampling stage; our earliest harvest occurred at early pod development (56 DAT), whereas [39] sampled throughout fruit maturation. Differences in cultivar, growing conditions, and harvest intervals may further explain the contrasting flavonoid accumulation patterns.

They further explained that the observation could be due to the stoppage or slow rate of new biosynthesis of phenolic compounds during the okra maturation. Hence, the okra size, firmness, and optimal harvest time may affect the phenolic compound concentration [39]. The DPPH and ORAC capacities were analyzed and compared across three harvest times (first, second, and third). The results showed significant differences (*p* < 0.05) in the second and third harvests for most treatments. In the second harvest, the DPPH values ranged from 7.72 ± 2.22 to 17.04 ± 1.93 mg TE/g DW, while in the third harvest, they ranged from 6.64 ± 0.17 to 14.58 ± 0.64 mg TE/g DW. Similarly, the ORAC values ranged from 9.07 ± 0.67 to 12.30 ± 0.53 µmol TE/g DW in the second harvest, and from 7.73 ± 0.42 to 14.71 ± 1.22 µmol TE/g DW in the third harvest. Although significant differences were observed among the harvest times, a general decrease in DPPH capacities was noted across treatments as the harvest period progressed throughout the growing season. A similar study, conducted by [39], showed that DPPH increased at the first stage of maturation and slightly decreased as the okra maturation stage extended throughout the growing season.

### 3.3. Ascorbic Acid (Vitamin C) and β-Carotene (Provitamin A)

Ascorbic acid (vitamin C), considered the most labile of all the vitamins found in food, can improve the utilization of iron and prevent several diseases, such as cancer, cataracts, type 2 diabetes, and cardiovascular diseases, due to its antioxidant and antihistamine effects [9]. Studies have shown that oxidation reactions, light, oxygen, and heat quickly degrade ascorbic acid. Ascorbic acid is lost during the processing, cooking, and storage of green leafy vegetables [9]. Vitamin C retention was used as a marker to investigate the effect of different drying methods, such as Reactance Window and freeze-drying, on asparagus, which is also a highly perishable vegetable [40].

Vitamin A is a fat-soluble vitamin that is also easily degraded by heat and oxidation when exposed to air. Provitamin A can be found in green and yellow fruits and vegetables [41]. Vitamin C in organic okra was 22.5 mg/100 g before boiling, while beta-carotene was about 10 mg/100 g (fresh weight) after blanching [42]. The results of the ascorbic acid (vitamin C) and β-carotene (provitamin A) content of the different pre-treated and dried okra samples from the second harvest are shown in Table 6. No significant differences were observed in vitamin C and alpha-carotene among the treatments. The results show that the vitamin C content tends to decrease with certain pre-treatments and drying methods, but these changes were not significant (*p* > 0.05), indicating that the treatments did not have a measurable effect on vitamin C under the conditions tested. In a study conducted by [43], they evaluated the thermal performance, bioactive compounds, antioxidant activity, and functional properties of okra. Their results were not directly comparable with the current study, as they found that the vitamin C content degraded due to the drying method. They stated that many contributing factors, such as processing methods, temperature, and the sample’s physical properties, may cause degradation. Significant differences were observed in provitamin A content (beta-carotene and total carotene) among the treatments (Table 6). All thermal pre-treatments (steam- and hot water-blanched) increased the measured β-carotene and total carotenoid contents relative to the control. This increase is likely attributable to improved extractability and thermal-induced isomerization rather than an actual increase in the total carotenoid content in the samples, indicating that the observed differences reflect higher measurable, extractable carotenoids rather than a true increase in total carotenoid levels. Based on the drying kinetics of this study, it was observed that the control samples took longer to dry, which may have contributed to the degradation of vitamin A in those samples [9]. Similar results to the current study were shown in a study that evaluated the effects of pre-treatment methods on the proximate, sensory properties, and vitamin compositions of okra [41].

In accordance with the current results, the changes in the content of carotenoids and vitamin E during tomato thermal processingcan sometimes exceed the measured β-carotene content due to enhanced extractability [44]. Similar trends have been reported in tomato processing studies, where thermal treatment can sometimes result in a higher measured β-carotene content compared with fresh samples. This increase is generally attributed to the enhanced extractability and isomerization of carotenoids following heat-induced disruption of the plant matrix. Thus, higher β-carotene levels observed after blanching and drying reflect improved availability of carotenoids. Their findings are comparable to the current results, as we also observed an increase in β-carotene following the application of thermal processing. The results of the current study are also consistent with those reported by [45], who found that carotenoid concentrations were much higher in canned tomato samples compared to raw tomatoes. However, this increase may be partially attributed to technological treatments such as pasteurization and homogenization applied during canning, which can improve the extractability of pigments from the vegetable.

## 4. Conclusions

The effects of the processing and preservation methods on the nutritional quality of okra varied significantly across harvest stages. Because thermal pre-treatments and drying methods influenced individual nutritional components differently, no single processing combination optimized all quality attributes. Therefore, processing strategies should be selected based on specific nutritional outcomes of interest. For example, steam-blanching followed by freeze-drying generally retains more phenolics and flavonoids, whereas other treatment combinations are better for certain vitamins.

Overall, each thermal and drying treatment investigated had different effects on the components examined, making it difficult to specify the ideal thermal process and drying treatment that will provide optimal preservation of the nutritional values of okra. For the majority of the parameters evaluated in this study, freeze and hot air-drying performed comparably and tended to retain higher nutrient levels than infrared-drying under the conditions evaluated. These findings indicate that the choice of processing methods should balance nutrient retention with practical considerations such as cost, processing capacity, and appearance of dried value-added products.

Despite the expense and the duration of drying, the freeze-drying method was shown to be the most effective method for the value-added production and preservation of phytonutrients and vitamins. The results from the current study also suggested that the hot air-drying method could be used as an economical alternative for developing dried okra value-added products. It is noted that high temperatures commonly associated with the hot air-drying method often cause deterioration in important nutrients and color, loss of flavor, and a decrease in the rehydration ability of the final products. Infrared-dried samples generally showed slightly lower retention of TPC, TFC, and β-carotene compared with freeze-dried and, in some cases, hot air-dried samples. For example, C-ID samples in the first harvest had 6.53 ± 0.3 mg GAE/g TPC, which was lower than C-FD (7.92 ± 0.3 mg GAE/g) and slightly higher than C-HAD (5.42 ± 0.2 mg GAE/g). These results suggest that, under the conditions used in this study, infrared-drying may be less efficient than freeze-drying in preserving phenolic compounds.

In conclusion, the results indicate a substantial (*p* < 0.05) influence of processing and preservation methods and harvest times on the antioxidant activity and phenolic, flavonoid, and vitamin contents of okra. Overall, steam-blanching followed by freeze-drying (SB-FD) provided a good compromise between phenolic and flavonoid retention of and enhancement in β-carotene content. While unblanched controls often exhibited the highest TPC, TFC, and DPPH values, hot water-blanched treatments generally showed higher β-carotene, and hot air-dried samples occasionally matched or exceeded infrared-dried samples for ORAC and DPPH. These results highlight that the effectiveness of pre-treatment and drying methods depends on the specific bioactive compound measured, and no single combination was universally superior across all parameters.

Limitations and Future Study: This study was limited to a single okra cultivar grown during one growing season at a single location, and vitamins were measured only for the second harvest. Additionally, no sensory, color, or long-term storage stability data were collected, which restricts the interpretation of product quality beyond the measured bioactive and phytonutrient traits. Future research should evaluate multiple cultivars across different environments, assess sensory and color attributes, and investigate the stability of vitamins, phenolics, and antioxidant capacity during extended storage to better inform the development of dried okra value-added products.

## Figures and Tables

**Table 1:** Average moisture content (%) and water activity (a_w_) of okra as affected by thermal pre-treatments and drying processes.

Treatment		Moisture Content (%)	Water Activity (a_w_)
Thermal	Drying		
Control	Freeze-Dried	6.72	0.35
	Hot Air-Dried	5.41	0.35
	Infrared-Dried	4.73	0.39
Steam-blanched	Freeze-Dried	5.10	0.29
	Hot Air-Dried	3.18	0.44
	Infrared-Dried	4.60	0.39
Hot water-blanched	Freeze-Dried	6.29	0.31
	Hot Air-Dried	3.16	0.39
	Infrared-Dried	3.76	0.29
SE		1.20	0.065

Standard error (SE). n = 3.

**Table 2:** Total phenolic content in dried okra (mg GAE/g DW) as affected by thermal pre-treatments, drying processes, and harvest times.

Treatment		Harvest Times		
Thermal	Drying	1st Harvest	2nd Harvest	3rd Harvest
Control	Freeze-Dried	7.92 ± 0.3 ^aA^	8.23 ± 0.9 ^aA^	8.62 ± 0.4 ^aB^
	Hot Air-Dried	5.42 ± 0.2 ^cB^	8.25 ± 0.5 ^aA^	5.54 ± 0.1 ^bB^
	Infrared-Dried	6.53 ± 0.3 ^bA^	6.21 ± 0.2 ^bA^	4.09 ± 0.2 ^cdB^
Steam-blanched	Freeze-Dried	4.77 ± 0.2 ^cdeB^	5.53 ± 0.3 ^bcA^	3.76 ± 0.3 ^dB^
	Hot Air-Dried	4.67 ± 0.3 ^deB^	5.50 ± 0.5 ^bcA^	3.65 ± 0.1 ^dB^
	Infrared-Dried	4.59 ± 0.1 ^eB^	4.89 ± 0.1 ^cA^	4.10 ± 0.1 ^cdB^
Hot water-blanched	Freeze-Dried	6.65 ± 0.4 ^bA^	5.04 ± 0.1 ^bcB^	3.65 ± 0.2 ^dC^
	Hot Air-Dried	5.35 ± 0.2 ^cdB^	5.83 ± 0.2 ^bcA^	4.48 ± 0.2 ^cB^
	Infrared-Dried	4.95 ± 0.3 ^cdeA^	4.10 ± 0.3 ^cA^	3.85 ± 0.1 ^cdB^

Harvest times = 1st (56 DAT), 2nd (84 DAT), 3rd (114 DAT); GAE = milligram of gallic acid equivalents per gram of sample; DW, sample dry weight basis. Data are means ± SD values. ^a–e^ Means with different letters within the same column indicate significant (*p* < 0.05) differences between treatments. ^A–C^ Means with different upper-case letters within the same row indicate significant (*p* < 0.05) differences based on harvest times.

**Table 3:** Total flavonoid content in dried okra (mg CE/g DW) as affected by thermal pre-treatments, drying processes, and harvest times.

Treatment		Harvest Times		
Thermal	Drying	1st Harvest	2nd Harvest	3rd Harvest
Control	Freeze-Dried	2.32 ± 0.03 ^aA^	1.87 ± 0.1 ^abcB^	1.87 ± 0.1 ^bB^
	Hot Air-Dried	2.26 ± 0.03 ^aA^	1.94 ± 0.1 ^abB^	2.47 ± 0.1 ^aA^
	Infrared-Dried	2.12 ± 0.1 ^abB^	2.17 ± 0.1 ^aAB^	2.30 ± 0.01 ^aA^
Steam-blanched	Freeze-Dried	1.22 ± 0.03 ^dB^	1.78 ± 0.03 ^bcdA^	1.42 ±c 0.3 ^AB^
	Hot Air-Dried	1.49 ± 0.03 ^cdA^	1.77 ± 0.3 ^bcdA^	1.09 ± 0.1 ^dB^
	Infrared-Dried	1.55 ± 0.01 ^cdB^	1.69 ± 0.01 ^bcdA^	1.44 ± 0.1 ^cC^
Hot water-blanched	Freeze-Dried	2.00 ± 0.3 ^abA^	1.52 ± 0.04 ^dB^	1.11 ± 0.03 ^dB^
	Hot Air-Dried	2.06 ± 0.1 ^abA^	1.97 ± 0.04 ^abA^	1.32 ± 0.05 ^cdB^
	Infrared-Dried	1.86 ± 0.1 ^bcA^	1.54 ± 0.1 ^cdB^	1.43 ± 0.04 ^cB^

Harvest times = 1st (56 DAT), 2nd (84 DAT), 3rd (114 DAT). CE, (+)-catechin equivalents; DW, sample dry weight basis. Data are means ± SD values. ^a–d^ Means with different letters within the same column indicate significant (*p* < 0.05) differences between treatments. ^A–C^ Means with different upper-case letters within the same row indicate significant (*p* < 0.05) differences based on harvest times.

**Table 4:** DPPH radical scavenging capacity (mg TE/g DW) in dried okra as affected by thermal pre-treatments, drying processes, and harvest times.

Treatment		Harvest Times		
Thermal	Drying	1st Harvest	2nd Harvest	3rd Harvest
Control	Freeze-Dried	16.44 ± 0.78 ^aA^	16.23 ± 1.64 ^abA^	11.31 ± 0.39 ^bB^
	Hot Air-Dried	15.58 ± 0.67 ^aA^	17.04 ± 1.93 ^aA^	14.58 ± 0.64 ^aA^
	Infrared-Dried	13.03 ± 0.94 ^bAB^	12.10 ± 0.15 ^bcB^	14.52 ± 0.75 ^aA^
Steam-blanched	Freeze-Dried	9.61 ± 0.22 ^dB^	11.30 ± 0.34 ^cA^	7.13 ± 0.42 ^cC^
	Hot Air-Dried	9.60 ± 0.35 ^dA^	10.43 ± 1.52 ^cA^	6.67 ± 0.28 ^cB^
	Infrared-Dried	8.33 ± 1.32 ^dA^	8.92 ± 0.28 ^cA^	7.06 ± 0.14 ^cA^
Hot water-blanched	Freeze-Dried	12.01 ± 0.42 ^bcA^	7.72 ± 2.22 ^cB^	7.55 ± 0.73 ^cB^
	Hot Air-Dried	9.65 ± 0.37 ^cdB^	11.35 ± 0.69 ^cA^	7.98 ± 0.21 ^cC^
	Infrared-Dried	7.82 ± 0.17 ^dA^	8.38 ± 0.21 ^cA^	6.64 ± 0.17 ^cB^

Harvest times = 1st (56 DAT), 2nd (84 DAT), 3rd (114 DAT). TE, Trolox equivalents; DW, sample dry weight basis. Data are means ± SD values. ^a–d^ Means with different letters within the same column indicate significant (*p* < 0.05) differences between treatments. ^A–C^ Means with different upper-case letters within the same row indicate significant (*p* < 0.05) differences based on harvest times.

**Table 5:** Oxygen radical absorbance capacity (µmol TE/g DW) in dried okra as affected by thermal pre-treatments, drying processes, and harvest times.

Treatment		Harvest Times		
Thermal	Drying	1st Harvest	2nd Harvest	3rd Harvest
Control	Freeze-Dried	4.96 ± 0.38 ^bC^	10.71 ± 0.78 ^abB^	13.00 ± 0.53 ^abA^
	Hot Air-Dried	4.26 ± 0.23 ^bB^	11.38 ± 1.55 ^abA^	14.71 ± 1.22 ^aA^
	Infrared-Dried	4.16 ± 0.09 ^dC^	12.30 ± 0.53 ^aB^	14.91 ± 0.83 ^aA^
Steam-blanched	Freeze-Dried	4.03 ± 0.11 ^bB^	10.29 ± 0.70 ^abA^	8.92 ± 0.88 ^cdeA^
	Hot Air-Dried	4.06 ± 0.32 ^bB^	9.77 ± 1.23 ^abA^	7.73 ± 0.42 ^eA^
	Infrared-Dried	9.39 ± 0.29 ^aAB^	9.90 ± 0.37 ^abA^	8.57 ± 0.21 ^deB^
Hot water-blanched	Freeze-Dried	10.08 ± 0.93 ^aA^	9.07 ± 0.67 ^bA^	9.13 ± 0.45 ^cdeA^
	Hot Air-Dried	9.46 ± 0.62 ^aB^	11.86 ± 0.22 ^abA^	10.98 ± 0.34 ^bcA^
	Infrared-Dried	9.50 ± 0.57 ^aA^	9.57 ± 0.35 ^abA^	10.25 ± 0.58 ^cdA^

Harvest times = 1st (56 DAT), 2nd (84 DAT), 3rd (114 DAT). TE, Trolox equivalents; DW, sample dry weight basis. Data are mean ± SD values. ^a–e^ Means with different letters within the same column indicate significant (*p* < 0.05) differences between treatments. ^A–C^ Means with different upper-case letters within the same row indicate significant (*p* < 0.05) differences based on harvest times.

**Table 6:** Ascorbic acid (vitamin C) (mg/100 g DW) and β-carotene (provitamin A) content µg RAE/100 g DW of the second harvest (84 DAT) okra as affected by thermal pre-treatments and drying processes.

Treatment		Vitamin C	Total	Total	Alpha
Thermal	Drying		β-Carotene	Carotene	Carotene
Control	Freeze-Dried	110.10 ^a^	40.40 ± 10.4 ^b^	41.10 ± 10.5 ^b^	0.17 ± 0.2 ^a^
	Hot Air-Dried	52.03 ^a^	39.80 ± 23.6 ^b^	41.13 ± 24.4 ^b^	0.03 ± 0.0 ^a^
	Infrared-Dried	47.23 ^a^	40.63 ± 10.6 ^b^	40.83 ± 10.6 ^b^	0.22 ± 0.3 ^a^
Steam-blanched	Freeze-Dried	32.90 ^a^	112.90 ± 28.6 ^a^	112.87 ± 29.6 ^a^	0.86 ± 0.3 ^a^
	Hot Air-Dried	91.80 ^a^	124.07 ± 22.5 ^a^	124.90 ± 23.7 ^a^	0.94 ± 0.5 ^a^
	Infrared-Dried	70.30 ^a^	134.33 ± 14.8 ^a^	135.00 ± 14.4 ^a^	0.60 ± 0.2 ^a^
Hot water-blanched	Freeze-Dried	54.80 ^a^	147.33 ± 15.6 ^a^	148.00 ± 15.0 ^a^	1.02 ± 0.5 ^a^
	Hot Air-Dried	74.90 ^a^	147.00 ± 10.0 ^a^	147.33 ± 10.1 ^a^	0.87 ± 0.3 ^a^
	Infrared-Dried	42.43 ^a^	120.70 ± 29.0 ^a^	120.37 ± 28.5 ^a^	0.40 ± 0.6 ^a^

RAE—retinol activity equivalents. Data are mean ± SD values. ^a,b^ Means with different letters within the same column are significantly different (*p* < 0.05).

## Data Availability

The original contributions presented in the study are included in the article, further inquiries can be directed to the corresponding authors.

## Abbreviations

The following abbreviations are used in this manuscript:

C	Control
SB	Steam-blanched
HWB	Hot water-blanched;
FD	Freeze-dried
HAD	Hot air-dried
ID	Infrared-dried
RAE	Retinol activity equivalents
DAT	Days after transplant
TE	Trolox equivalent
DW	Dry weight
ORAC	Oxygen radical absorbance capacity
CE	(+)-catechin equivalents
GAE	Milligram of gallic acid equivalents
TPC	Total phenolic content
TFC	Total flavonoid content
DPPH	1,1 dipheny1-2-picrylhydrazyl radical scavenging capacity
